# Polythiophene and oligothiophene systems modified by TTF electroactive units for organic electronics

**DOI:** 10.3762/bjoc.11.191

**Published:** 2015-09-28

**Authors:** Alexander L Kanibolotsky, Neil J Findlay, Peter J Skabara

**Affiliations:** 1 WestCHEM, Department of Pure and Applied Chemistry, University of Strathclyde, 295 Cathedral Street, Glasgow, G1 1XL, United Kingdom; 2 Institute of Physical-Organic Chemistry and Coal Chemistry, 02160 Kyiv, Ukraine

**Keywords:** donor, oligothiophene, organic electronics, polythiophene, semiconductor, tetrathiafulvalene

## Abstract

The aim of this review is to give an update on current progress in the synthesis, properties and applications of thiophene-based conjugated systems bearing tetrathiafulvalene (TTF) units. We focus mostly on the synthesis of poly- and oligothiophenes with TTF moieties fused to the thiophene units of the conjugated backbone either directly or via a dithiin ring. The electrochemical behaviour of these materials and structure–property relationships are discussed. The study is directed towards the development of a new type of organic semiconductors based on these hybrid materials for application in organic field effect transistors and solar cells.

## Introduction

Sulfur-rich π-functional systems are important building blocks in materials chemistry. Among them, tetrathiafulvalene (TTF) electron donor and polythiophene (PT) conjugated systems are highly popular classes of organic compounds which have shown fascinating conducting and electronic properties. The advantages of PT-based materials are their synthetic versatility, excellent film-forming properties and potential to increase the dimensionality of charge carrier transport [1] by involving π–π stacking interactions. Varying the substituents of the conjugated backbone allows control over the polymer’s effective conjugation length and electronic properties, whilst also influencing the extent of inter-chain interactions. Two of the most studied polythiophene materials for organic electronics are regioregular poly(3-hexylthiophene) (P3HT) [2–3] and poly(3,4-ethylenedioxythiophene) (PEDOT) [4], which is highly conductive in its doped state. P3HT has become a benchmark polymer semiconductor for both bulk hetero-junction solar cells (BHJSCs) [5] and organic field effect transistors (OFETs) [6–7], whereas the PEDOT:poly(styrene sulfonate) salt (PEDOT:PSS) was originally investigated for antistatic applications but is now commercially available for its use as a hole-injecting/collecting material for organic light emitting diodes (OLEDs) and BHJSCs. So far, various electroactive units have been anchored aside the polythiophene backbone, including ferrocene [8], porphyrin [9], 2-carboxyanthraquinone [10], 1,3-dithiole-2-ylidenefluorene [11–12], dithiinoquinoxaline [13–14] and fullerene C_60_ [15–16]. The incorporation of acceptor units into a conjugated network is a standard way to narrow the HOMO/LUMO band gap and examples of such units include dioxopyrrolopyrrole (DPP) [17–19], benzodifuranone [20] and boron-dipyrromethene (BODIPY) [21–22].

As a different class of electroactive materials, TTF derivatives are well-known as reversible redox systems with low potentials of oxidation to cation radical and dication species. The high level of stability observed for the oxidised TTF π-electron system arises from the aromatic nature of the oxidised 1,3-dithiolium rings and this has triggered tremendous efforts directed toward the synthesis of compounds with TTF donor units and subsequent investigation of their properties. Since the first discovery of the semiconducting properties of TTF and its cation radical [23], and the metallic behaviour of the TTF-TCNQ charge transfer complex [24], great attention was focused on the preparation of TTF mixed valance state materials, which showed superconducting properties [25]. Fusing the TTF unit with dithiin rings in bis(ethylenedithio)tetrathiafulvalene (BEDT-TTF) led to the extension of 1D π–π stacking intermolecular interactions in a donor sheet of a mixed valance state system to 2D with a significant contribution from S···S non-covalent interactions [26]. This gave a record transition temperature among TTF mixed valence ambient pressure superconductors in the salt κ-(BEDT-TTF)_2_Cu[N(CN)_2_]Br [27]. In an attempt to create macromolecular compounds with multi-electron redox activity and to further increase the dimensionality of their intermolecular interactions in the solid phase, the TTF units were incorporated into dendritic structures [28–32]. The extraordinary propensity of TTF and its doped species to aggregate was the reason for using this unit in the design of gelators [33–34].

Combining the exceptional donor strength of TTF and excellent film-forming properties of a conjugated polymer (CP) opens up the possibility to create promising materials with interesting redox behaviour. So far the TTF unit has been used for redox modification of various CP systems [35] including incorporation within the conjugated backbone [36–37], as a pendant unit [38–40] and direct fusion to the π-conjugated system of the polymer [41–42]. Incorporation of a TTF unit into a PT architecture allows the creation of interesting hybrid redox systems with a wide range of electro-activities. The goal of this review is to provide an update on the synthesis of TTF-PT hybrid conjugated systems, their properties and their application to organic electronics. Both electrodeposition and chemical polymerisation will be considered as methods of producing the PT conjugated backbone. In some cases poly(ethynylene/vinylene) homologues will be considered for comparison. Additionally, monodispersed tetrathiafulvalene-oligothiophene (TTF-OT) conjugated systems will be discussed as their well-defined structures provide a stronger insight into structure–property relationships.

## Review

### PT conjugated systems with TTF units within the polymer backbone or as pendant units

**Scheme 1:**
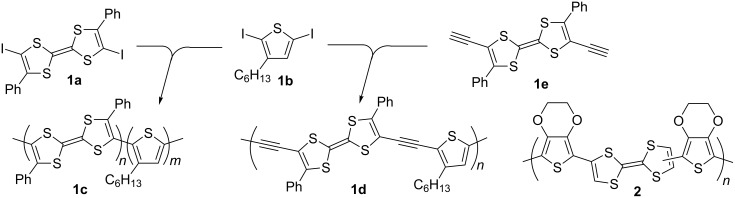
The synthesis of PT based conjugated systems with the TTF unit incorporated within the polymer backbone.

The most straightforward way to modify PT conjugated systems is to incorporate the TTF unit into the polymer backbone or attach it as a pendant unit, as only minor modifications to the synthesis of the TTF/thiophene monomer are required. Both chemical [43] and electrochemical polymerisation [44] have been used to incorporate a TTF moiety within the polythiophene backbone. Yamamoto coupling of diiodo monomers **1a** and **1b** provided polymer **1c**, albeit with a modest molecular weight (*M*_w_ = 5800 Da) compared to that of the polymer **1d**, which was obtained by Sonogashira coupling of **1b** with **1e** and exhibited a partial solubility in THF with *M*_w_ = 610000 Da (THF soluble fraction) [43] (Scheme 1).

Polymer properties in the solid state are hugely important for organic electronics applications, with the electronic properties of materials being greatly affected by film morphology. The electropolymerisation technique creates a simple test for the viability of a certain structural motif in the PT chain and is considered both as a potential route for the synthesis of a new functional CP and also as a method for the modification of an electrode surface [45]. The electrodeposition of a polymer has a number of advantages over any chemical protocol: 1) it is cheap and can be performed on a very small scale; 2) it requires no reagent or catalyst and is very clean; 3) due to the interfacial nature of a polymer growth the spectro-electrochemical investigation of the polymer film is straightforward; 4) it provides control over the morphology through the choice of solvent, electrolyte and/or the method of electrodeposition. TTF has been incorporated into a CP backbone by electropolymerisation of its bis(EDOT) derivative to afford polymer **2** [44] (Scheme 1). All the polymers (**1c,d** and **2**) exhibited electro-activity of the TTF units. Due to a break in conjugation of the polymers in their neutral state there is no electrochemical signature of the PT backbone. As such, the aforementioned systems cannot be considered as true TTF-PT systems.

**Scheme 2:**
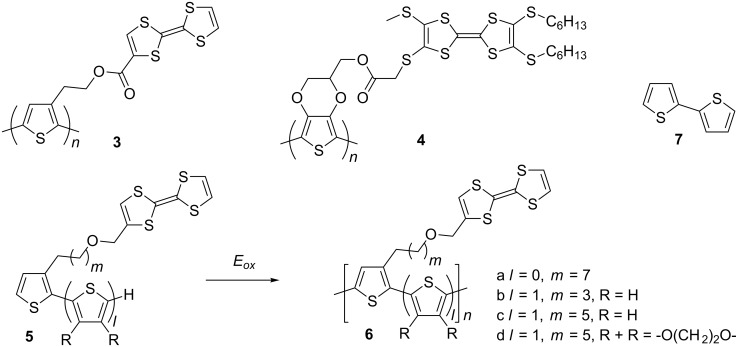
PT with pendant TTF units, prepared by electropolymerisation.

The first example of the electrochemical preparation of PT with a pendant TTF-carboxylic unit (**3**) was reported by the Bryce group [46] (Scheme 2). The mixture of CH_2_Cl_2_ with PhNO_2_, to supress the electrochemical activity of TTF during electropolymerisation, was used as a solvent. Another example of a TTF-PT hybrid polymer (**4**), now with an ester linkage between the tetrathio-TTF derivative and the PEDOT polymer backbone, has also been reported [47]. The TTF-functionalised EDOT monomer unit allowed the authors to manage the electropolymerisation in an acetonitrile:CH_2_Cl_2_ mixture using both potentiodynamic and potentiostatic electrodeposition. Nevertheless, the labile ester bond and its potential cleavage remain an issue due to formation of acid upon electropolymerisation.

**Figure 1:**
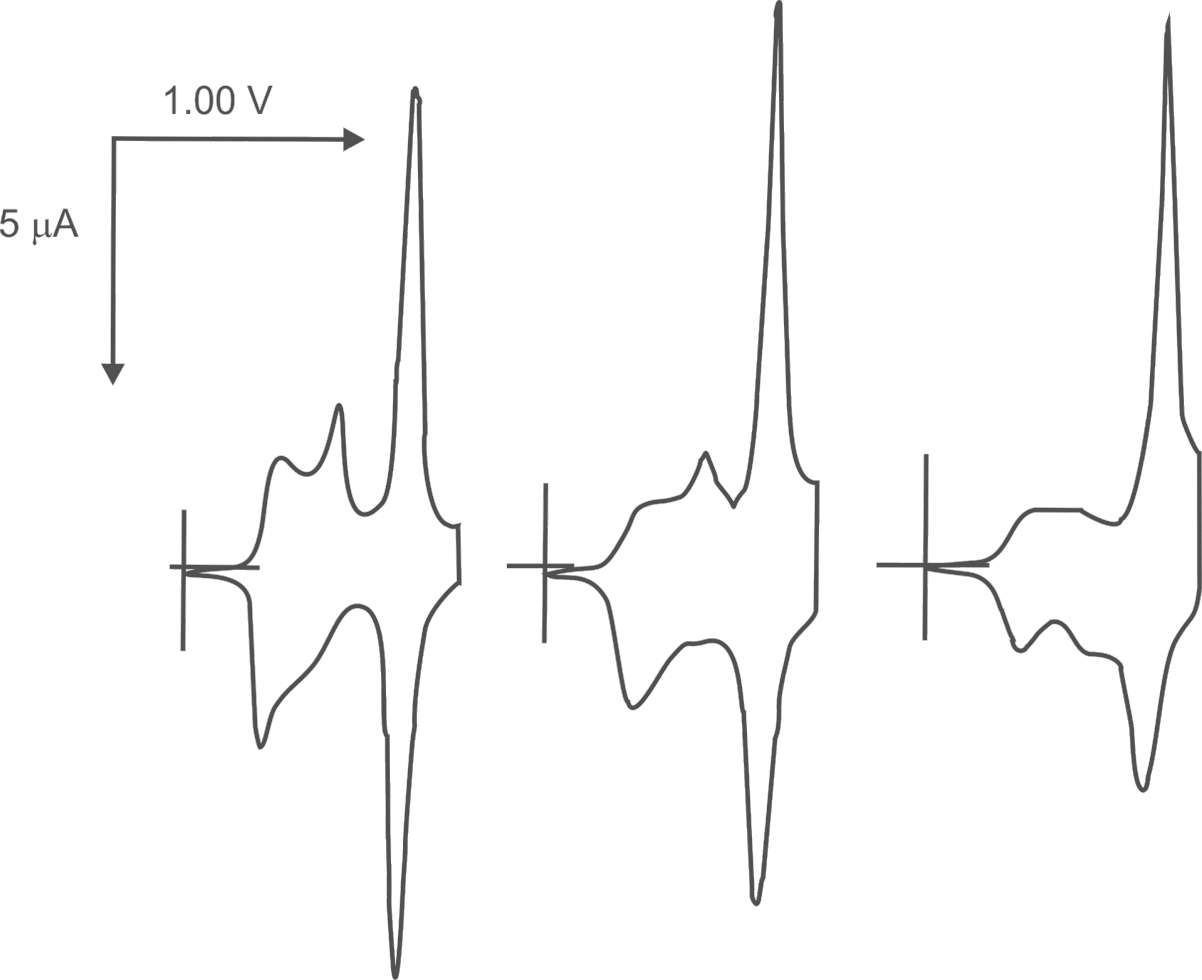
Cyclic voltammograms of copolymers electrodeposited from nitrobenzene solutions of TTF modified monomer **6c** and nonsubstituted bithiophenes **7**. Left: 2 × 10^−2^ M of **6c**; middle: 2 × 10^−2^ M of **6c** + 1 × 10^−2^ M of **7**; right: 2 × 10^−2^ M of **6c** + 4 × 10^−2^ M of **7**; ref. SCE, 0.1 M (TBA)PF_6_ in acetonitrile as an electrolyte. Reproduced with permission from [49]. Copyright 1998 Wiley-VCH.

Roncali and co-workers used more reliable ether bonds to anchor a TTF moiety to a thiophene monomer via a long aliphatic spacer group, avoiding the effect of steric interactions between pendant TTF units and increasing the conjugation length of the PT backbone (Scheme 2). They successfully electropolymerised TTF-modified thiophene monomer **5a** to polymer **6a** from a nitrobenzene monomer solution [48]. Cyclic voltammetry of the polymer thin film revealed the splitting of the first oxidation wave during the cathodic run, which the authors attributed to a stepwise reduction from the aggregated radical cation to an intermediate mixed valence state, then further reduction to the neutral species. To decrease the difference in the oxidation potential of TTF and that of the thiophene backbone of the monomer, the TTF-modified bithiophene compounds **5b–d** were used as monomers for electropolymerisation to **6b–d** [49]. The appearance of an additional, well-defined oxidation wave in the CV, as the first oxidation wave was split in both anodic and cathodic runs, was evident for all polymers, but clearest for **6c**. This was assigned to the formation of a mixed valence state and aggregated cation radical [50]. The relative increase in the peak current during oxidation to the dication, compared to that of cation radical formation, was explained by an additional contribution to charge transport from the doped PT backbone [49]. The oxidation of the latter did not contribute significantly to the CV of the polymer films due to the much stronger electrochemical response of the TTF. However, from a separate experiment in which the authors electropolymerised monomer **5c** (2 × 10^−2^ M) in the presence of a double excess of a non-modified bithiophene monomer **7** [49], the contribution from the PT backbone oxidation in the CV of the final copolymer was clear. However, it was unresolved from the wave of TTF^2+^ formation during the anodic run (Figure 1).

**Scheme 3:**
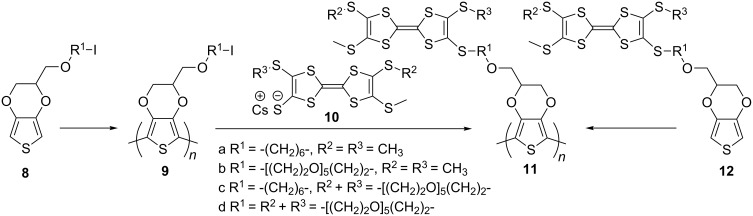
PT with pendant TTF units prepared by electropolymerisation and post-modification of polymerised PT through iodoalkyl functionality.

An alternative way of preparing PT hybrid materials with TTF pendant groups was to modify the pre-polymerised PT containing an appropriate functionality with a TTF derivative. The Roncali group reported electropolymerisation of EDOT monomers **8a,b** bearing a ω-iodo-functionalised aliphatic chain to polymers **9a,b**, which was followed by the heterogeneous reaction of the polymeric film with TTF thiolates **10a** [51] and **10c** [52] to produce the polymers **11a–d** (Scheme 3). The polymers **11a,c** were also prepared by electropolymerisation of the corresponding TTF functionalised monomers **12a,c** [52]. The electrochemical response from the polymeric film of **11c**, prepared by functionalising prepolymerised PEDOT **9a** with thiolatoTTF **10c**, and that prepared by direct electropolymerisation of **12c** turned out to be very similar, confirming that the heterogeneous derivatisation of **9a** with **10c** was rapid and quantitative with no significant effect on the integrity of the polymer. The crown ether TTF modified polymers **11c,d** were tested for electrochemical recognition of Ba^2+^ ions. At [Ba^2+^] saturation concentration of 4 mM the shifts of the first CV peak for **11c** and **11d** films were +60 and +30 mV with a TTF electrode coverage of 1.4 × 10^−9^ and 9 × 10^−9^ mol/cm^2^, respectively.

**Scheme 4:**
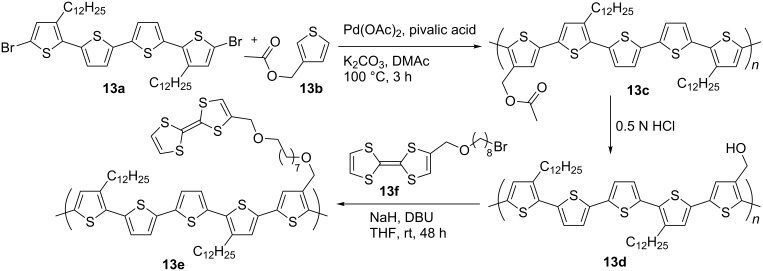
Synthesis of PT with pendant TTF by post-modification of the polymer prepared by direct arylation.

A recent example of the chemical preparation of PT with pendant TTF-units has been reported [53] (Scheme 4) where direct arylation polymerisation of quaterthiophene **13a** and 3-(acetoxymethyl)thiophene (**13b**), followed by acidic hydrolysis of the ester groups in polymer **13c**, provided the polymer **13d** with hydroxy groups for further modification by ω-bromooctyloxymethylTTF **13f** . The CV of the final PT-TTF compound **13e** showed mainly the characteristics of the PT backbone; due to the low content of the TTF unit in the polymer **13e**, the two oxidation waves related to formation of TTF cation radical and dication were not apparent in the CV of the film, but were discernible in solution state. Pure **13e**, and **13e** with a small amount of the parent poly(3,3'''-didodecyl-2,2':5',2'':5'',2'''-quaterthiophene) (PQT12) (5 or 10 wt %), did not exhibit any OFET activity due to hole trapping by the TTF unit. This hole trapping was explained to be the reason for a negative Seebeck coefficient of the non-doped polymer **13e** and was used for sensing trinitrotoluene (TNT) using the drain-source current-increase response to TTF-TNT complexation in an OFET fabricated from **13e** with 5% of PQT12.

### Conjugated OT systems with fused TTF units

#### Synthesis of the monomer units

**Scheme 5:**
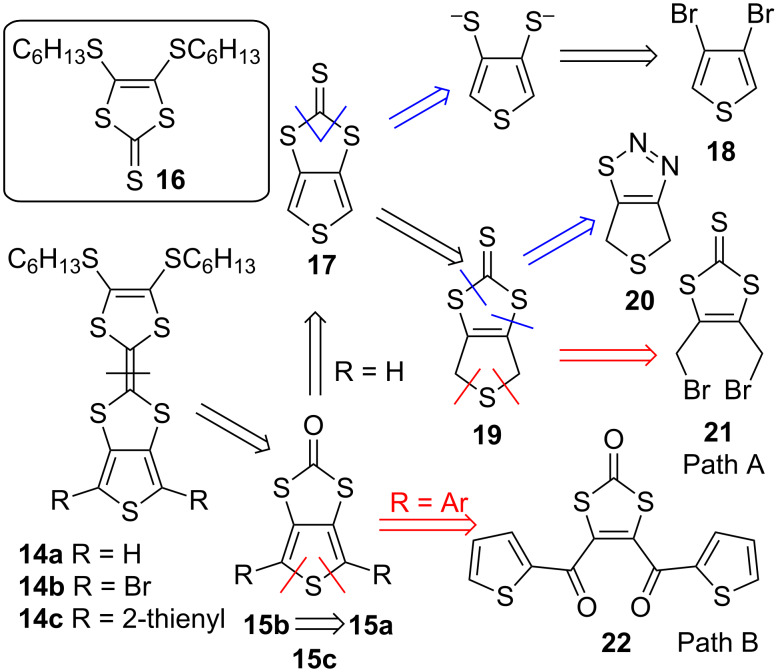
Retrosynthetic scheme for the synthesis of the monomer building block which is required for the preparation of PT with TTF directly fused to the polymer backbone. Bis(bromomethyl) derivative **21** and diketone **22** are starting compounds for synthetic pathways A and B, respectively.

Incorporation of a TTF unit into a PT architecture via fusion to the polymer backbone allows the realisation of highly diverse electroactive conjugated systems with different contributions to the properties from each of the components. In contrast to polymers where TTF is attached as a pendant unit or incorporated within a PT backbone, the construction of the TTF unit in this case is normally performed through coupling of the corresponding dithiol units, with one or both of them already fused to the monomer thiophene backbone precursor. The retrosynthetic scheme for these monomers with direct fusion of the TTF unit to a thiophene **14a–c** is shown in Scheme 5, with the key building block thieno[3,4-*d*][1,3]dithiole-2-one **15a–c**.

Where there is no substitution at the α-position of the thiophene monomer, e.g., **14a**, triethylphosphite mediated heterocoupling of **15a** with 4,5-bis(hexylthio)-1,3-dithiole-2-thione (**16**) proceeds in low yield (20–30%) [54]. However, the same procedure for the synthesis of dibromo derivative **14b** turned out to be more effective, with the monomer **14b** being isolated in 70% yield [55]. The starting compound required for this, 4,6-dibromothieno[3,4-*d*][1,3]dithiole-2-one (**15b**), can be efficiently obtained by brominating compound **15a**, which in turn is synthesised by mercury(II) acetate assisted transchalcogenation reaction from the corresponding thione **17**. Unsubstituted thieno[3,4-*d*][1,3]dithiole-2-thione (**17**) can be constructed by building up either of its two rings, involving cyclisation of a suitable precursor already containing one existing heterocycle. The construction of the 1,3-dithiole-2-thione unit of **17** can be completed using 3,4-dibromothiophene (**18**) as a starting material [56], or by oxidation of dihydroderivative **19** obtained from 4,6-dihydrothieno[3,4-*d*][1,2,3]thiadiazole (**20**) [57–58]. However, the most reliable method for the synthesis of 4,6-dihydrothieno[3,4-*d*][1,3]dithiole-2-thione (**19**) is cyclisation of 4,5-bis(bromomethyl)-1,3-dithiole-2-thione (**21**) [59] (synthetic pathway A).

**Scheme 6:**
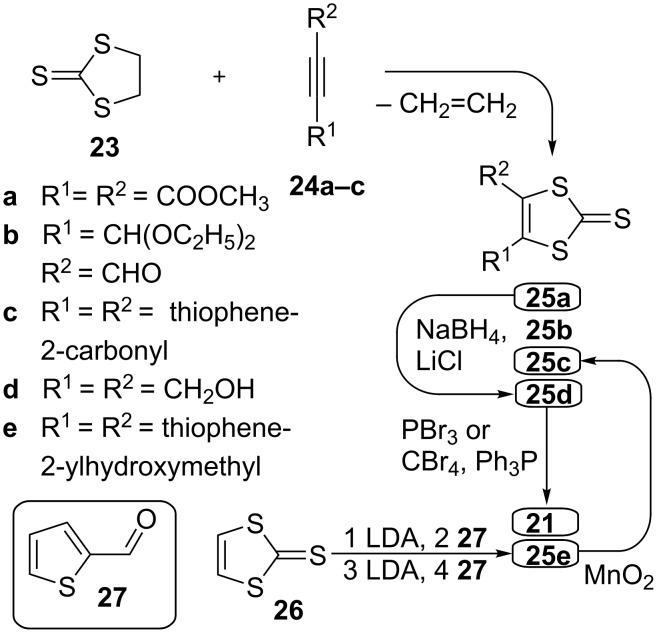
Synthesis of bisfunctionalised derivatives of vinylene trithiocarbonate **21** and **25c** required for synthetic pathways A and B, respectively.

For 4,6-diaryl substituted thienodithiole-2-ones, e.g., 4,6-di(thiophen-2-yl)thieno[3,4-*d*][1,3]dithiol-2-one (**15c**), construction of the thiophene directly onto the dithiole ring seems to be the only strategy, which can be readily achieved by reductive cyclisation of diketone **22** [60] (synthetic pathway B). Compound **22** is normally synthesised by transchalcogenation from the corresponding 1,3-dithiole-2-thione derivative. One method for the synthesis of 1,3-dithiole-2-thione with electron-acceptor substituents [61] is the reaction of readily accessible ethylene trithiocarbonate (**23**) [62] with electron-deficient acetylene compounds (Scheme 6). By reacting **23** with **24a** this method provides diester **25a** in good yield [63]. Compound **25a** can be reduced to diol **25d** [64] and further converted by either the Appel method [59] or by reaction with PBr_3_ [65] into dibromomethyl compound **21**, which is required for synthetic pathway A.

Even though the reaction of **23** with acetylene compound **24b** (containing only one electron-withdrawing group) is efficient, affording **25b** with a 60% yield [66], attempts to invoke cycloaddition of **23** and **24c** in a similar manner led to a poor yield of **25c** (8%) [60]. An efficient method for the synthesis of **25c** – a compound required for synthetic pathway B – was found to be repeated sequential lithiation of vinylene trithiocarbonate (**26**) [67] followed by subsequent trapping of the lithium organic species with thiophenecarboxaldehyde **27** [60]. The diol **25e**, formed as a product of this reaction, is unstable and undergoes various rearrangments [68–69] in acidic conditions. Hence, it is preferably oxidised directly to the more stable diketone **25c** without delay.

**Scheme 7:**
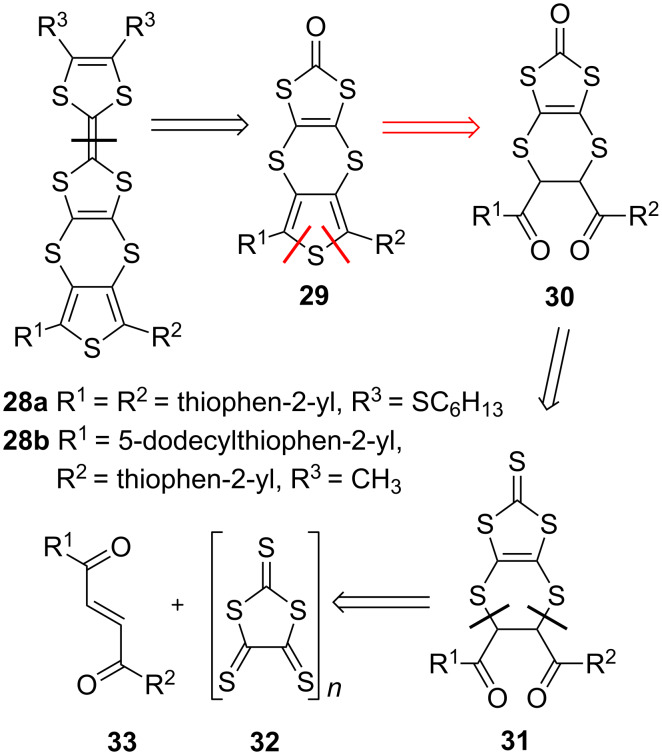
Retrosynthetic scheme for the synthesis of the building block which is required for the preparation of PT with TTF fused to a polymer backbone via a dithiin ring (synthetic pathway C).

The retrosynthetic scheme for the monomer units **28a,b** with thieno-dithiino-dithiole type fusion is shown in Scheme 7. Similar to the aforementioned synthetic pathway B, the strategy for the synthesis of **29** involves construction of the thiophene ring by cyclisation of diketone **30** (synthetic pathway C).

The diketone **31** is constructed through the cycloaddition reaction of diacylethene **33** with oligomer **32**, readily available by oxidation of bis(tetraethylammonium) bis(2-thioxo-1,3-dithiole-4,5-dithiolato)zincate with iodine [70]. This versatile strategy can be applied where R^1^ and R^2^ are either aromatic or aliphatic [71]. The application of the strategy has been utilised for both symmetric **28a** [60] and asymmetric **28b** systems [72].

#### Polymers with fused TTF units

The electronic characterisation for monomer units **14a–c** and **28a** is shown in Table 1.

**Table 1:** Electrochemical and UV–vis absorption data for the monomer compounds **14a–c**, and **28a** in CH_2_Cl_2_ solution. The oxidation potentials are shown vs Ag/AgCl reference.

Compound	*E*^1/2^_1ox_, V	*E*^1/2^_2ox_, V	*E*^p^_3ox_, V	λ_max_, nm

**14a**	0.74	1.10	2.18	324
**14b**	0.95	1.31	–	337
**14c**	0.64	1.02	1.55	373
**28a**	0.64	0.99	1.52	344

**Scheme 8:**
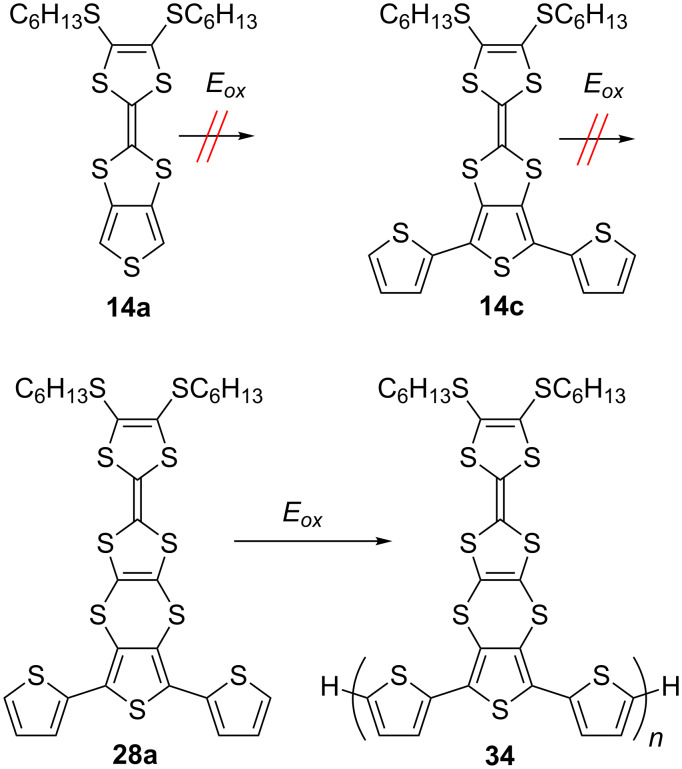
The monomers **14a**, **14c** and electropolymerisation of **28a**.

Electropolymerisation of monomer compounds **14a**, **14c** and **28a** [73] was attempted. Due to the high oxidation potential (see Table 1) of the thiophene unit in the fused system **14a** (2.18 V vs Ag/AgCl), electropolymerisation for this compound was unsuccessful (Scheme 8). Surprisingly, the other two monomers, both with a similar, low *E*^p^_3ox_ – attributed to oxidation of the terthiophene unit (**14c** (1.55 V vs Ag/AgCl) and **28a** (1.52 V vs Ag/AgCl)) – showed different behaviour upon repetitive voltammetric cycling over the range of 0.0–1.6 V vs Ag/AgCl. Although upon electrodeposition of **14c** onto the surface of a working electrode a red film appeared, it was non-polymeric in nature. On the contrary, the electropolymerisation of **28a** under the same conditions exhibited a reproducible polymer growth of **34**.

**Figure 2:**
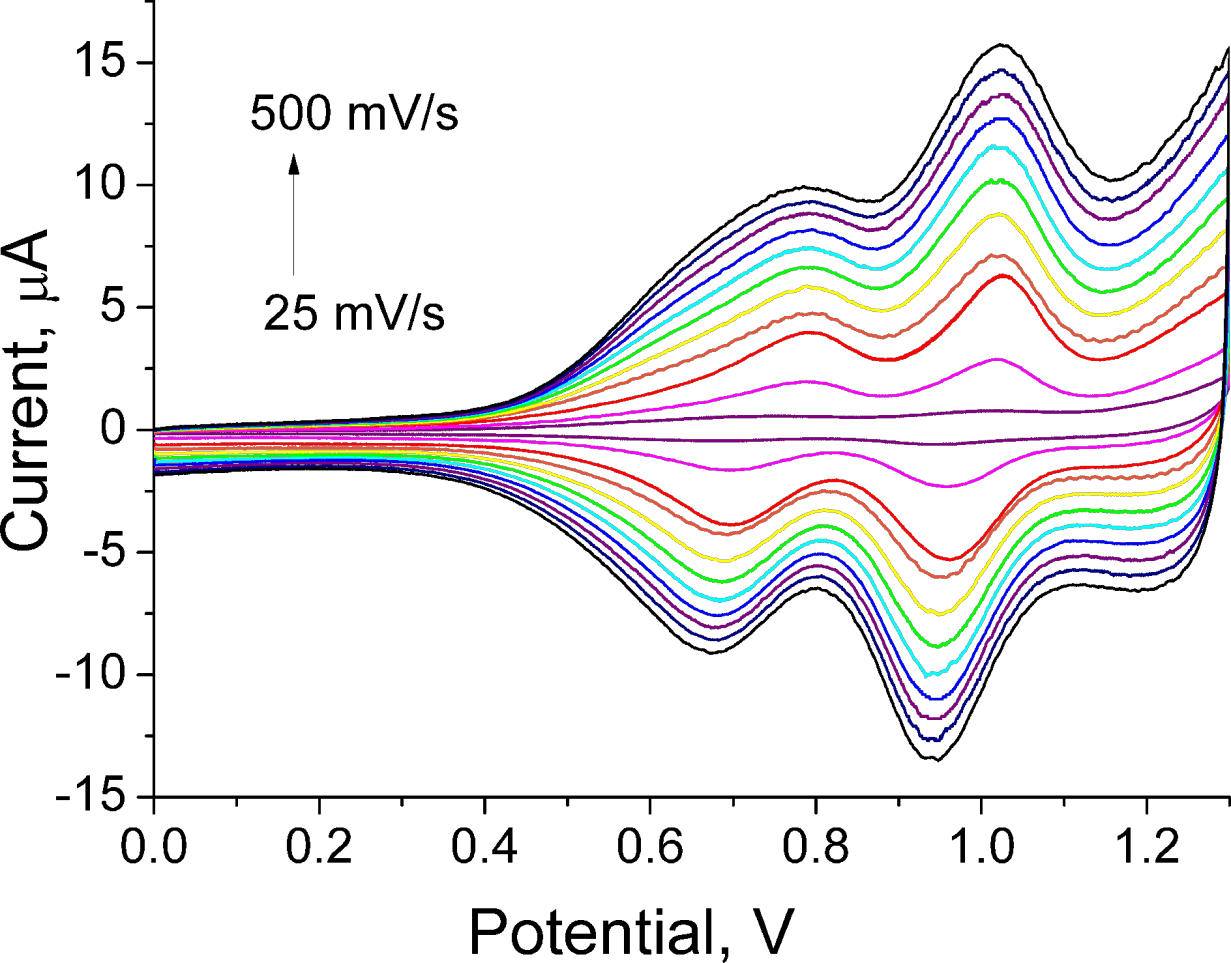
Cyclic voltammograms of a thin film of **34** at various scan rates (25 mV, 50 × *n* mV/s, *n* = 1–10). Adapted with permission from [73].Copyright 2000 The Royal Society of Chemistry.

The CV of polymer **34** exhibited the characteristic electrochemical signature of the TTF-unit – two reversible oxidation waves to a cation radical and a dication, with a linear dependence of peak currents upon scan rates. Similar to PT systems with a pendant TTF unit [49], the peak current of the second wave was noticeably higher than that of the first (Figure 2). There are three possible reasons for such behaviour that can be considered: 1) the interaction between the TTF units would cause the partial splitting of the first oxidation wave with poorly resolved components – attributed to oxidation of the neutral TTF into a mixed valence state and further to an aggregated cation radical; 2) contrary to formation of the cation radical, the oxidation to the dication is not limited by charge transport through the film as the conductivity of the latter is ensured by both charged TTF species and the polaron charge carrier route; 3) the oxidation potential of the polymer backbone is likely to be in the same region as the potential for TTF^2+^ formation, although the contribution to the current from the normally irreversible oxidation of the PT backbone would be small due to the high TTF content in the polymer.

The as grown polymer exhibited two broad absorption peaks at 459 and 833 nm, indicating that the polymer film exists in a doped state. The aforementioned peaks can be assigned to the cation radical of the TTF unit and are very similar to the absorption features of tetraalkylthiotetrathiafulvalene cation radical [31–32]. The doped film exhibited excellent stability and its absorption characteristics did not change despite treatment with hydrazine. However, de-doping was achieved with repetitive scanning of the polymer film over the range of −0.3–0 V vs Ag/AgCl for 2 hours [73]. After de-doping, the polymer **34** exhibited an absorption band with a maximum at ca. 490 nm and extending to ca. 736 nm, with an optical band gap of 1.69 eV (Table 2). For a simple π-conjugated polymer the difference between the oxidation and reduction onsets constitute the electrochemical band gap [74–75]. For polymer **34**, if the first oxidation wave was taken into consideration, the electrochemical band gap was found to be 1.39 eV. However, if the second oxidation wave was considered the band gap was calculated to be 1.81 eV, a value slightly higher than the aforementioned optical band gap of the polymer (1.69 eV). This is to be expected considering that upon oxidation of the PT backbone the electron must be removed from a polymer already containing oxidised TTF moieties. The agreement between the optical and electrochemical band gaps in this case infers that the oxidation of the PT backbone in the polymer **34** occurs at the potential close to the second oxidation wave of the TTF unit. A more detailed spectroelectrochemical study [76] of the polymer **34** film deposited on ITO glass revealed an electrochemical signature of both oxidised TTF species (TTF^+•^, TTF^2+^) [31–32] and polarons (vide infra).

**Table 2:** Characterisation of the polymers **34**, **35**, **37**, and **39**.

Polymer	*M*_n_, Da	PDI	Condition	*E*^1/2^_1ox_, V	*E*^1/2^_2ox_, V	*E*^pc^_1red_, V	λ_max_, nm (*E*_g_^opt^, eV)	
							CH_2_Cl_2_	Film

**34**	–	–	Film	0.77	1.09	−1.21	–	494 (1.69)
**35**	3437	1.32	CH_2_Cl_2_	0.81	1.10	−1.15	466(1.86)	487 (1.75)
**37**	4886	2.40	CH_2_Cl_2_	0.69	1.07	−1.65	456	496 (1.82)
**39**	3158	1.19	CH_2_Cl_2_	0.89	1.31	–	578	–
			Film	0.91^a^	1.35^a^	−0.96	–	598 (1.45)

^a^Due to the irreversible nature of the oxidation waves, the anodic peak values *E*^pa^_ox_ are shown.

**Scheme 9:**
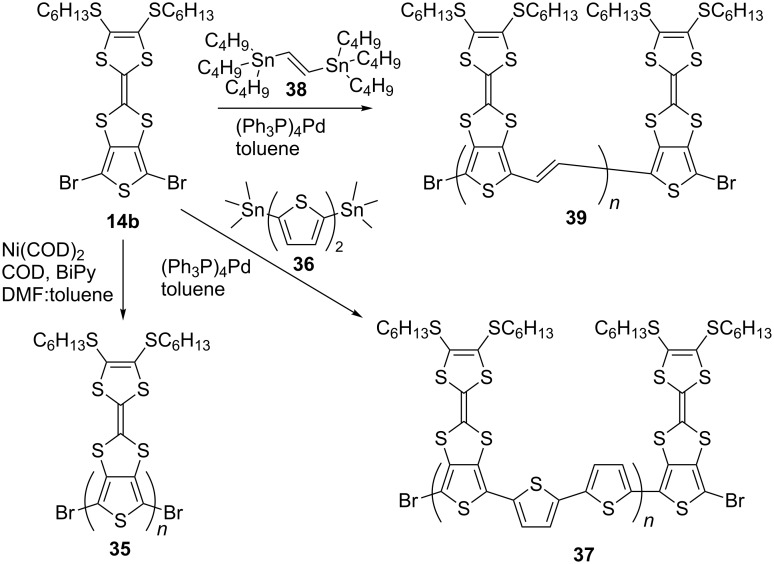
Chemical polymerisation of **14b** into polymers **35**, **37** and **39**.

To investigate the properties of PT-TTF systems with the TTF moiety directly fused to a thiophene backbone, chemical polymerisation of suitably functionalised monomers was carried out (Scheme 9). For all chemically synthesised polymers a Soxhlet extraction (with methanol, acetone and CH_2_Cl_2_) has been used as a method of purification and to narrow their polydispersity. Using Yamamoto coupling compound **14b** was polymerised. Use of DMF alone as solvent led to a polymer that was sparingly soluble in CH_2_Cl_2_ [77]. However, a mixture of DMF:toluene (1:1) as a medium for Yamamoto polymerisation afforded polymer **35** as a dark purple solid in 95% yield [55]. Polymer **35** is the analogue of the polymer which could have been obtained had the monomer **14a** been suitable for electropolymerisation, while Stille coupling of dibromo monomer **14b** with 5,5'-bis(trimethylstannyl)-2,2'-bithiophene (**36**) [78] was used to circumvent problems with the electropolymerisation of terthiophene **14c**, and to chemically synthesise the analogous polymer **37** [76]. By reacting monomer **14b** and 1,2-bis(tributylstannyl)ethylene (**38**) [79], polymer **39** was synthesised using the Stille coupling protocol [76].

The number average molecular weight (*M*_n_) revealed by GPC analysis (Table 2) corresponds to about 7 thienoTTF monomer units per polymer chain for **35** and **37**, and about 6 units for **39**. MALDI–TOF MS characterisation was only successful for polymer **39** and showed a series of peaks with a mass difference of 516 Da, corresponding to the mass of the 2-(4,5-bis(hexylthio)-1,3-dithiol-2-ylidene)thieno[3,4-*d*][1,3]dithiol-4,6-diyl-alt-vinylene repeating unit. The mass spectra confirmed that the polymer was end-capped with a thienoTTF unit, with the terminal bromo substituents still intact. The highest mass peak of 5290 Da registered by MALDI–MS corresponds to 10 thienoTTF units, which is significantly higher than the aforementioned *M*_n_ measured by GPC.

Table 2 displays the electrochemical and UV–vis absorption characteristics of the polymers. The peaks corresponding to the absorption maximum occurred in the range 450–500 nm for polymers **34**, **35** and **37**, with the optical band gap being in the range of 1.7–1.9 eV. When comparing the spectra in CH_2_Cl_2_ solutions to those of the solid film, the red shift in absorption is due to the emergence of π–π interactions in the solid state. Compared to the aforementioned polymers, poly(thienylenevinylene) (PTV) **39** exhibited a red-shifted absorption with maxima occurring at 578 and 598 nm in CH_2_Cl_2_ and as a thin film, respectively.

The CVs in CH_2_Cl_2_ solution of **35** and **37** (Table 2) revealed two quasi-reversible oxidation waves that are shifted to lower potentials compared to the corresponding reversible oxidation waves of monomer **14b** (+0.91 and +1.31 V, see Table 1). Monomer **14b** has a weaker donating ability due to the strong electron-withdrawing inductive effect of the terminal bromo substituents, while the PTV polymer **39** exhibited almost identical oxidation potentials to monomer **14b**. On the other hand, both oxidation waves of the TTF unit in polymers **35**, **37**, and **39** shifted to significantly higher potentials in comparison to those of the non-brominated monomer compound **14a** (+0.46 and +0.83 V, see Table 1) [54]. This can be explained by: 1) the electron-withdrawing effect of the polymer backbone and 2) the electrostatic interaction between the oxidised TTF units within the polymer backbone. The degree to which these polymer oxidation potentials shift is in the order **37** < **35** < **39**, which roughly follows the expected charge density of the doped polymer backbone. The chronocoulometry experiment during bulk electrolysis of **35** and **39** revealed that approximately two electrons were released per monomer unit; this is much more than one would expect from a simple PT that normally donates one electron per 3–10 thiophene units [80]. To the best of our knowledge, **35** is the most dopable polythiophene in the literature, with respect to the level of oxidation that is achieved per repeating unit, the excellent electrochemical reversibility observed, and the modest potential window in which the highly doped state is attained. Even for a stable doped system, for example a PEDOT sample heavily doped with polystyrenesulfonic (PSSH) or *p*-toluenesulfonic (TosH) acid, the doping level is 3–5 units per one positive charge [81–82]. So, the presence of TTF units fused to each thiophene of the PT backbone creates a polymer with a greatly enhanced p-doping ability. The direct fusion in this case of two electroactive units (TTF and PT) inhibits any electrochemical activity from the polymer backbone and the electrochemistry of the material is dominated by the TTF unit.

The inhibition of the polymer backbone’s electrochemical activity was confirmed by spectroelectrochemistry of **39**, which indicated no change of the π–π* transition upon applying potentials up to +2.0 V. The CV of **39** shows an irreversible first oxidation wave, and the band gap calculated from the first oxidation onset agreed well with the optical band gap. The former indicates the possibility of significant interchain interactions between the TTF unit and the polymer backbone.

**Figure 3:**
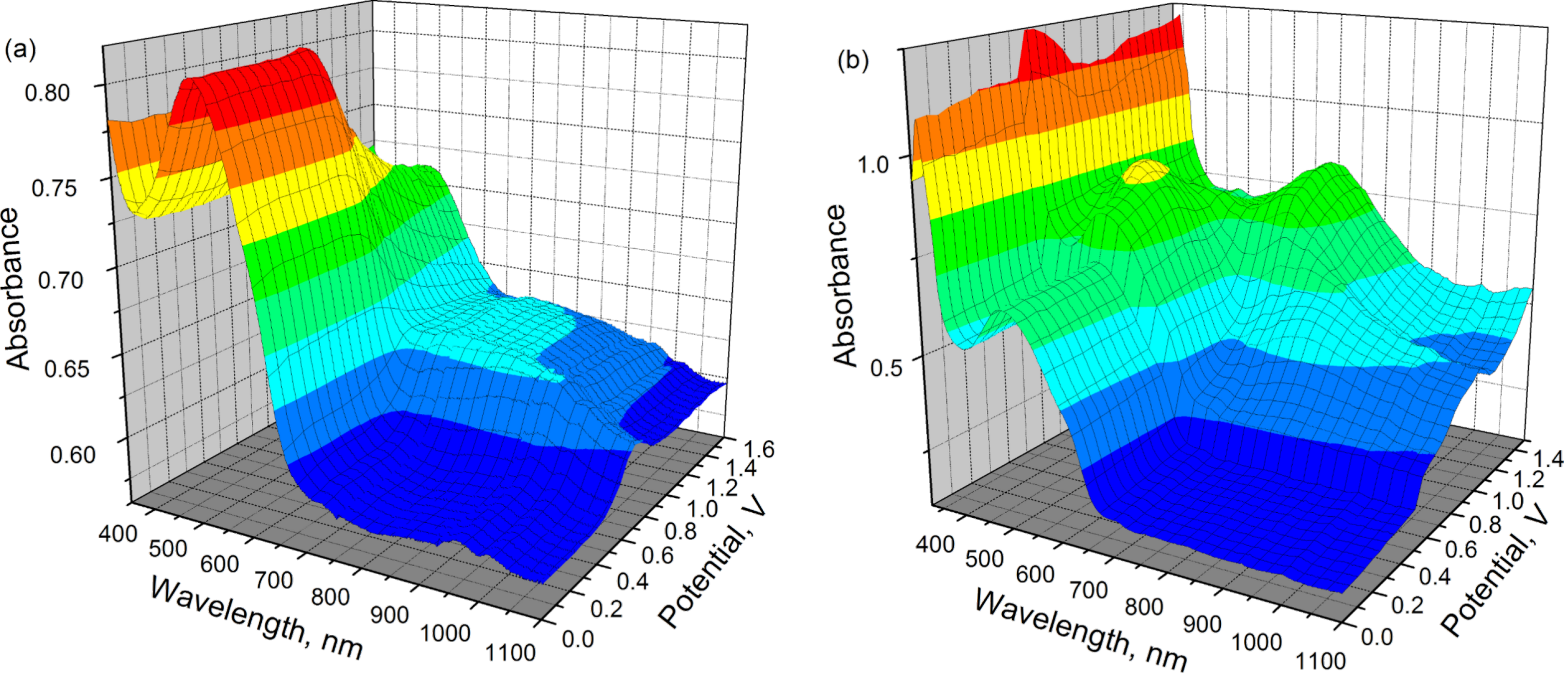
Spectroelectrochemistry of polymers **37** (a) and **34** (b) as thin films deposited on the working electrode. Adapted with permission from [76]. Copyright 2006 The American Chemical Society.

Upon oxidation, the film of polymer **37** exhibited a broad ill-defined band extending from 700 nm into the near infrared range. The intensity of the π–π* transition in this case diminished upon oxidation, but this band still remained the most intense feature of the spectrum across the entire potential range (Figure 3a). The spectoelectrochemistry of polymer **34** revealed more drastic changes in the spectra upon oxidation of the film, where the resolved absorption signature of a cation radical, dication and polaron can be observed (Figure 3b). As the applied potential is increased, two peaks appear: one at 460 nm that overlaps with the backbone π–π* transition, and a second centred at 800 nm. Those peaks were observed in the spectrum of the doped polymer film (Figure 3b) and could be assigned to the absorption of the cation radical of the TTF unit. With further increase in the applied potential the TTF cation radical UV–vis signature diminishes and a strong absorption band at 700 nm, along with a broad absorption in the NIR region, appear. The former can be assigned to the absorption of the dication of the TTF unit and both bands to a polaron formation. Therefore, for **34** the spectroelectrochemistry unequivocally confirms the electrochemical activity of the polymer backbone, which is involved in the formation of polarons at potentials close to the second oxidation potential of the TTF unit.

**Scheme 10:**
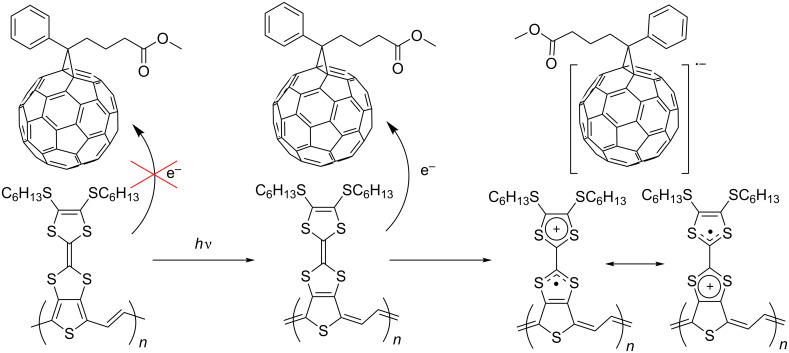
Photoinduced charge transfer from the TTF of polymer **39** to PC_61_BM.

The polymer **39**, which has the lowest band gap, was tested as a donor material for BHJSCs with PC_61_BM as the acceptor. The OPV performance is shown in Table 3. The estimation of HOMO and LUMO levels by electrochemical analysis gave values of −5.24 and −3.78 eV, respectively, indicating a small offset between the LUMO of **39** and PC_61_BM (ca. −3.8 eV) [83–84]. This is the main reason for the small short circuit current density (*J*_sc_) and low efficiency of the cell. Due to the possible presence of efficient interchain interactions between the TTF units and the PTV backbone (vide supra) in the film of polymer **39**, another important factor that should be considered is photoinduced charge transfer. Photoexcitation of **39** can lead to an increase in the donor ability of the TTF unit due to a greater contribution of the quinoidal structure to the excited polymer backbone, and foster electron transfer from the TTF moiety to PC_61_BM (Scheme 10). However, further hole transfer from the TTF unit to the PTV backbone may still limit dissociation of the (TTF^+•^)(PC_61_BM^−•^) bound pair.

**Table 3:** Performance of BHJSCs fabricated from the thiophene-TTF hybrid systems.

Compound	Acceptor	Solvent	*P*_inc_, mW cm^−2^	*J*_sc_, mA cm^−2^	*V*_oc_, V	FF	PCE, %

**39**	PC_61_BM	CB	80	0.68	0.52	0.30	0.13
**48**	none	o-DCB	100	1.8	0.61	0.28	0.31
**48**	PC_71_BM	CHCl_3_	100	4.9	0.66	0.31	1.0
**48**	PC_71_BM	o-DCB	100	8.0	0.71	0.32	1.8
**54** (*n* = 1)	PC_71_BM	CHCl_3_	100	7.44	0.70	0.33	1.7
**54** (*n* = 1)	PC_71_BM	o-DCB	100	9.81	0.78	0.33	2.5

**Scheme 11:**
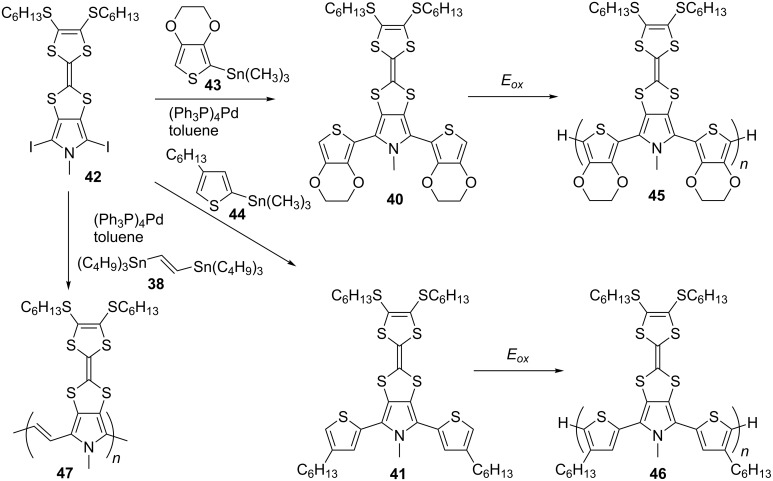
Electropolymerisation of **40** and **41** into polymers **45** and **46**, respectively, and Stille polymerisation of **42** into polymer **47**.

The surprising inertness of **14c** towards electropolymerisation can be circumvented by replacing the thiophene units in the monomer terthiophene backbone with more electron-rich moieties, such as pyrrole and EDOT. Bisthienylpyrrolo-TTF monomer compounds **40** and **41**, which were synthesised by Stille coupling of diiodopyrrolo-TTF **42** with trimethylstannyl derivatives of EDOT **43** and hexylthiophene **44**, were efficiently electropolymerised (Scheme 11) into polymers **45** and **46**, respectively [85]. Note that these latter two polymers are analogues of polymer **37**. Polymer **47**, synthesised by Stille polymerisation of **42** and **38**, is an analogue of the polymer **39**. A direct comparison between the obtained polymers with pyrroloTTF and thienoTTF units showed that the incorporation of an electron-rich pyrrole unit into the conjugated backbone leads to materials with a wider band gap as they are less stable to n-doping. The pyrrole unit lowers the oxidation potentials of the TTF moieties but the electrochemical dominance of the TTF is lost in the pyrrolo-TTF polymers.

**Scheme 12:**
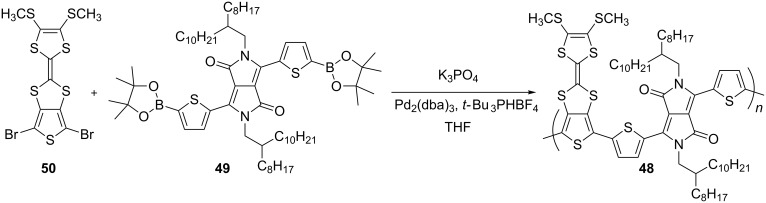
The synthesis of polymer **48**.

Another analogue of polymer **37** includes the 2,5-bis(2-octyldodecyl)-1,4-dioxopyrrolo[3,4-*c*]pyrrole (DPP) unit incorporated within the PT backbone [86]. The polymer **48** was prepared by Suzuki coupling polymerisation of diboronic ester **49** and dibromothieno-TTF **50** (Scheme 12). The latter was synthesised following the aforementioned synthetic pathway A. The incorporation of the DPP π-acceptor into the conjugated backbone led to a polymer with a narrow optical band gap (*E*_g_^opt^ = 1.32 eV in CH_2_Cl_2_ solution), with the expected lower value of *E*_g_^opt^ = 1.26 eV in the film due to π–π stacking interactions. The value of the HOMO/LUMO levels (−5.13/−3.49 eV) in the film were noticeably different from those in solution (−4.95/−3.55 eV), which suggested significant donor–acceptor interactions in the solid phase between the DPP and TTF units.

**Figure 4:**
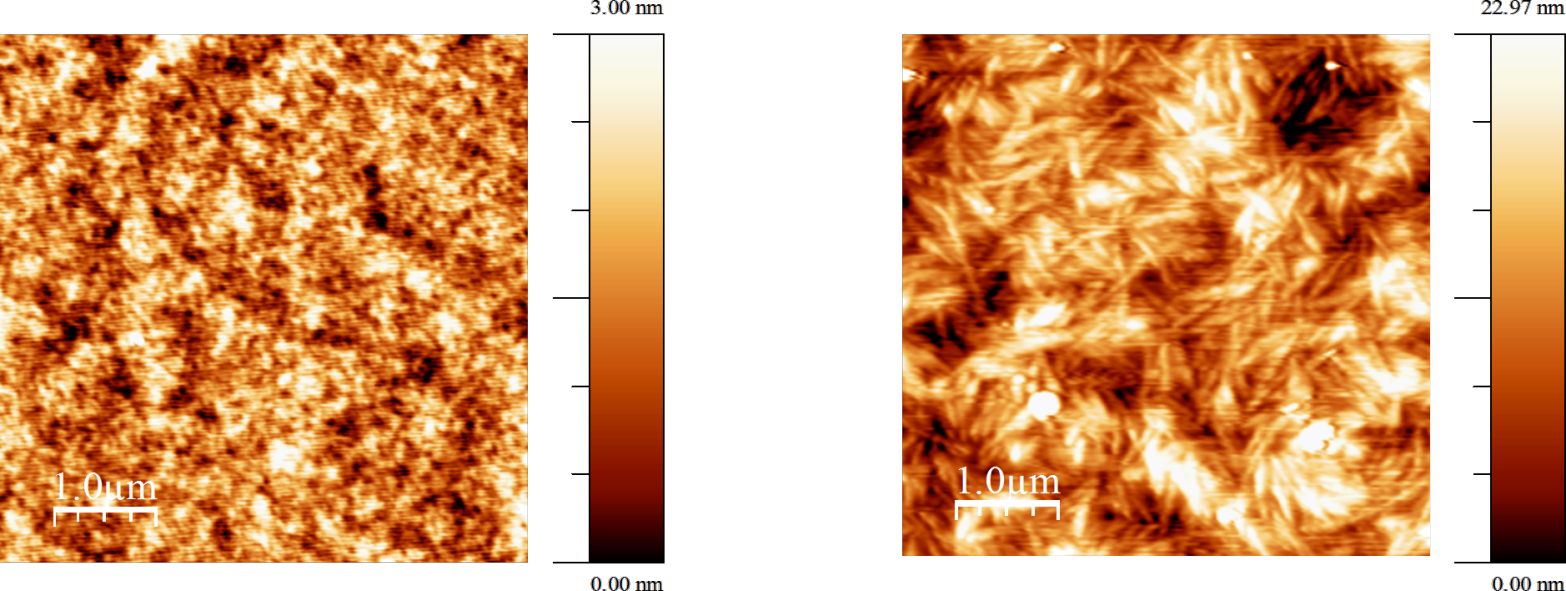
Tapping mode AFM height images of polymer **48** film spin-coated from chlorobenzene (left) and chloroform (right) solutions on ODTS treated SiO_2_ substrate. Reproduced with permission from [87]. Copyright 2015 The American Chemical Society.

OFET device fabrication employing polymer **48** exhibited p-type semiconductor behaviour, with the best performance from devices using the bottom contact top gate configuration [87]. The hole mobility values calculated in the saturated region were found to be 3.8 × 10^−2^ and 5.3 × 10^−2^ cm^2^ V^−1^ s^−1^ for OFETs fabricated via spincoating the semiconductor from solution in chlorobenzene and chloroform, respectively. The strong propensity of **48** to aggregation led to the tightly packed grain morphology of the film cast from chlorobenzene with small sized crystalline domains (Figure 4). On the contrary, using chloroform the high solvation energy of the TTF unit and the carbonyl groups of the DPP moieties made the rate of nucleation lower compared to the rate of grain growth, so the size of the crystalline domain in the film was higher in this case. These larger crystalline domains in films spin-coated from chloroform were beneficial for field effect mobility.

None of the OFETs showed any n-type mobility. The extended character of the HOMO residing on the dithienyl-thieno-TTF unit and the localised nature of the LUMO led to donor–acceptor interactions in the solid phase, making it impossible for efficient overlap between LUMOs, which would normally be required for an efficient n-type semiconductor.

BHJSCs were fabricated from **48** as the electron donor and PC_71_BM as the electron acceptor using *ortho*-diclorobenzene (*o*-DCB) and chloroform as solvents (Table 3). The devices prepared with *o*-DCB showed up to a two-fold increase in power conversion efficiency (PCE) compared to those obtained by spincoating the blend from chloroform, which is due to a more homogeneous blend morphology leading to improved charge carrier transport and increased *J*_sc_. Since the use of *o*-DCB as the solvent for spincoating provided better performance for BHJSCs than chloroform, it was used for the fabrication of a single material organic solar cell (SMOSC) (Table 3). The SMOSC performance is modest compared to that of similar devices fabricated using donor–acceptor block copolymers [88–90]. Nevertheless, the value of the PCE (0.31%) is higher than one would expect from a SMOSC fabricated from polymer **48** as a semiconductor, since it has no obvious donor–acceptor phase separation and is lacking efficient electron mobility.

#### TTF-oligothiophene systems with well-defined structures

**Scheme 13:**
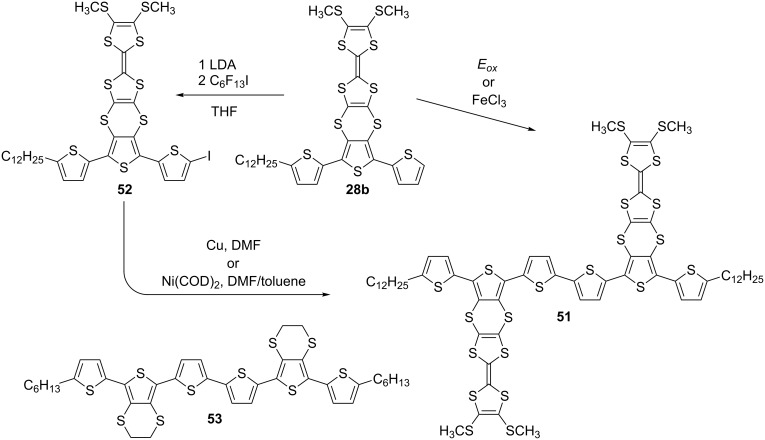
The synthesis of TTF-sexithiophene system **51** and the structure of the parent sexithiophene **53**.

The monodisperse analogue of polymer **34**, bearing two TTF units and capped with dodecyl chains at the terminal positions, was synthesised using chemical coupling protocols, or alternatively via electrochemical oxidation of terthiophene **28b** (Scheme 13) [72]. The latter was synthesised by the aforementioned synthetic pathway C.

The electrochemical method for the preparation of sexithiophene **51** was achieved by potentiostatic oxidative electrodimerization of **28b** in a mixture of 2:1 CH_2_Cl_2_/hexane, with 0.1 M tetrabutylammonium hexafluorophosphate as the supporting electrolyte. On a larger scale, chemical oxidation by FeCl_3_ in nitrobenzene was used which after purification, afforded **51** in a 24% yield. Lithiation of compound **28b** with LDA and successive trapping of the aryllithium compound with perfluorohexyl iodide afforded iodoterthiophene **52** in a 74% yield. Compound **52** was used to explore other possibilities for synthesising sexithiophene **51**, including Ullmann and Yamamoto coupling, which provided **51** in 43 and 10% yield, respectively. Sexithiophene **51** exhibited a strong propensity to aggregate even in chloroform solution, hence an interpretable ^1^H NMR spectrum was only obtained in a mixture of CDCl_3_ with CS_2_.

In CH_2_Cl_2_ solution, the chemically synthesised product showed a π–π* transition peak at 443 nm, with a HOMO–LUMO gap of 2.32 eV, a value very similar to that of the parent sexithiophene **53** [91]. For the electrochemically prepared film of **51**, there were two broad bands with maxima centred at 449 and 735 nm, confirming the doped state of the film and the presence of a cation radical centred on the TTF unit. After de-doping, a single broad band remained with its maximum red-shifted compared to that of the solution state spectrum of the chemically synthesised sexithiophene **51**; this is evidence of a strong π–π stacking interaction upon aggregation in the solid state. The electrochemistry of compound **51** is similar for both solution and solid state samples, with the main feature being the overlap between the second oxidation peak of the TTF and the oxidation of the sexithiophene backbone.

**Scheme 14:**
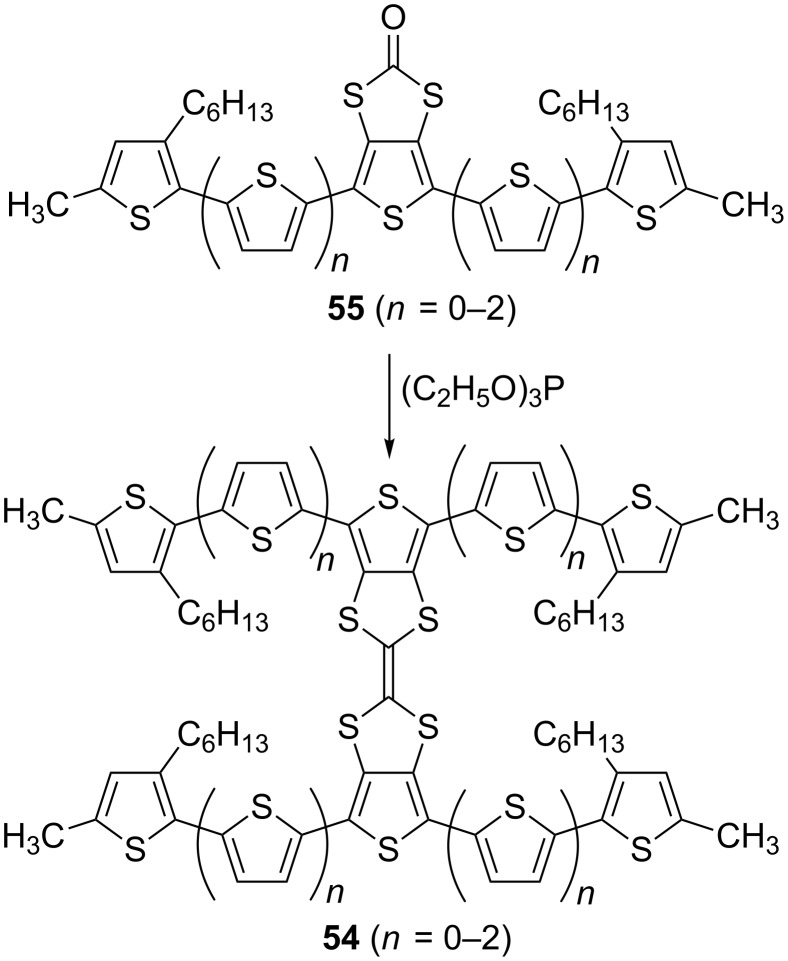
The synthesis of TTF-oligothiophene H-shaped systems **54** (*n* = 0–2).

A series of hybrid electroactive compounds **54** (*n* = 0–2) with two oligothiophenes directly fused to one TTF unit was recently reported [92–93]. Here, triethylphosphite mediated homo-coupling of the corresponding oligothiophenes **55** (*n* = 0–2) containing a central thieno[3,4-*d*][1,3]dithiole-2-one unit proceeded smoothly, with yields of 77% for *n* = 0, 82% for *n* = 1 and 39% for *n* = 2 (Scheme 14).

The oligothiophene half-unit precursors, **55** (*n* = 0–2), were synthesised following synthetic pathway B. The electrochemical and optical properties of **54** (*n* = 0–2) are shown in Table 4.

**Table 4:** The properties of monodisperse oligothiophene-TTF systems in dichloromethane solution.

	**51**	**54** (*n* = 0)	**54** (*n* = 1)	**54** (*n* = 2)

*E*_1ox_, V^a^	+0.29/0.21	+0.39/+0.32	+0.27/+0.21	+0.26/+0.23
*E*_2ox_, V^a^	+0.53/0.45	+0.86/+0.75	+0.54/+0.48	+0.66/+0.49
*E*_3ox_, V^a^	–	+1.13	+0.76/+0.71	+0.97/+0.94
*E*_4ox_, V^a^	–	–	+0.97/+0.89	–
*E*_red_, V^a^	–	–2.12	–2.19	−1.98
HOMO^b^, eV	−4.93	–5.06	–4.96	−4.95
LUMO^b^, eV	–	–2.92	–2.81	−3.00
HOMO–LUMO gap, eV	–	2.14	2.15	1.95
λ_max_, nm	443	351	431	461
Absorption onset, eV	2.32	2.92	2.45	2.20

^a^The electrochemical data are referenced against the Fc/Fc^+^ couple. Both *E*^pa^ and *E*^pc^ or anodic peak value (*E*^pa^, if the wave is irreversible) for the oxidation waves and cathodic peak values (*E*^pc^) for the reduction waves are quoted. ^b^HOMO/LUMO values were calculated using the formula HOMO/LUMO = −*E*^onset^_ox_/*E*^onset^_red_ − 4.80.

**Scheme 15:**
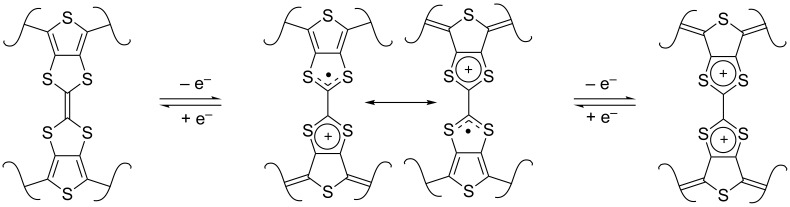
The oxidation of a fused TTF-oligothiophene system.

The optical properties of **54** (*n* = 0–2) in solution (Table 4) follow a general trend of decreasing the absorption onset, while increasing the conjugation length. The electrochemistry of each H-shaped system **54** (*n* = 0–2) on the other hand is not so straightforward. While the first and the second oxidation waves are easily identified and assigned for **51** (vide supra), for **54** (*n* = 0–2) it is only the first oxidation wave which can be unequivocally assigned to the first oxidation potential of the TTF unit. It is interesting to note that the terthiophene-TTF H-shaped system **54** (*n* = 0) exhibits a significantly higher potential for the formation of TTF^+•^ than that of the quinqui- and septithiophene systems **54** (*n* = 1–2). This can be explained by the increased π-donating ability of the oligothiophene with a higher conjugation length due to a more pronounced contribution of the quinoidal resonance structure. This effect is only possible for hybrid systems with a TTF unit directly fused to the PT backbone (Scheme 15).

Quinqui- and septithiophene TTF bridge systems **54** (*n* = 1–2) are prone to strong aggregation in solution and, as with **51**, a mixture of chloroform with CS_2_ was used for NMR spectroscopy in these cases. For compound **54** (*n* = 2) the absorption spectrum as a thin film exhibits a maximum at 496 nm and is red-shifted by 35 nm compared to the solution state spectrum, suggesting strong intermolecular interactions in the solid phase.

**Figure 5:**
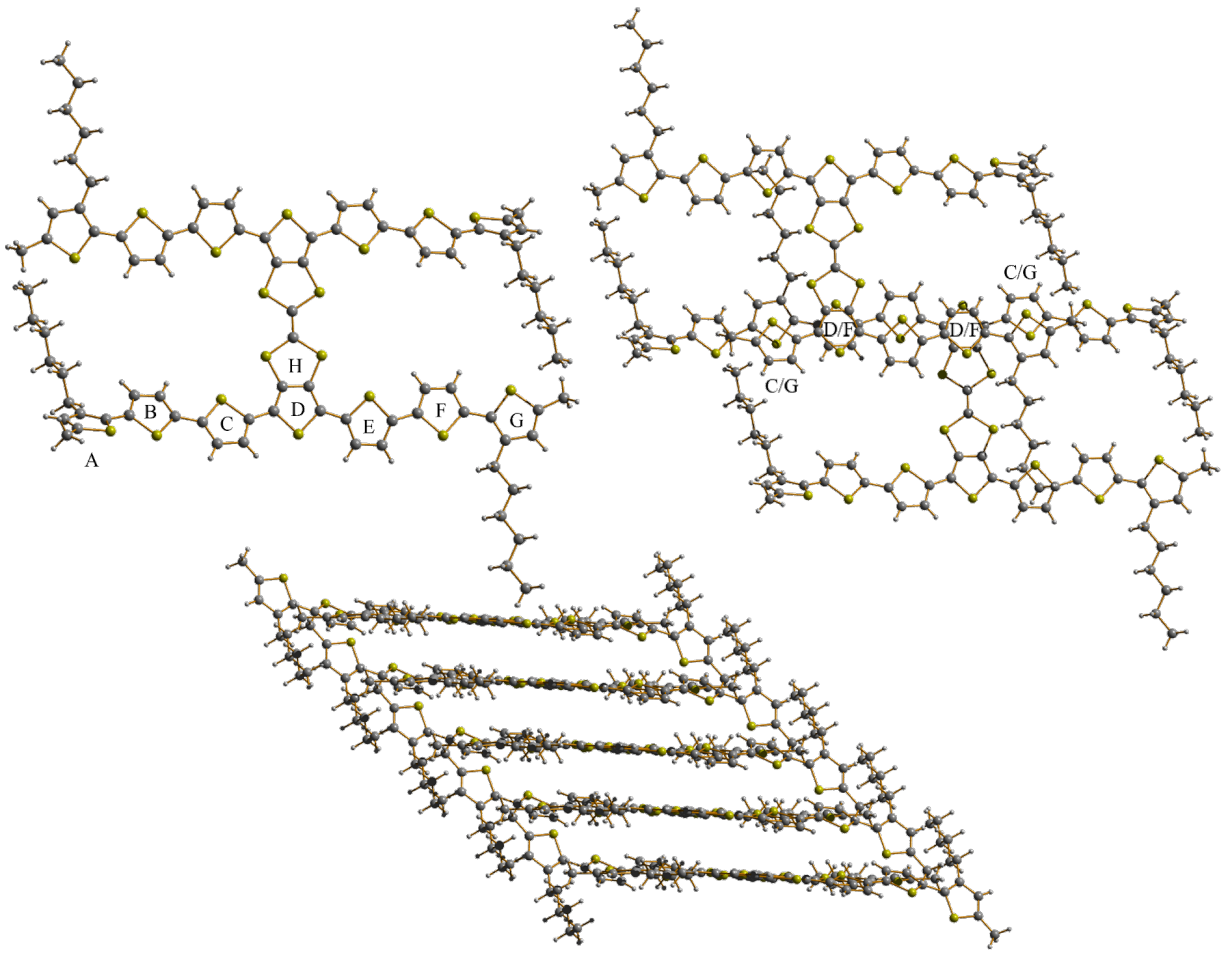
Molecular structure and packing arrangement of compound **54** (*n* = 2). Adapted by permission from [92]. Copyright 2011 The Royal Society of Chemistry.

Single crystal X-ray diffraction of **54** (*n* = 2) (Figure 5) revealed that the molecules in the solid state are essentially planar, apart from a significant torsion angle of 33° between the terminal thiophene A and the thiophene B.

The angle between planes D and F is 5.79°, with the inter-ring distance being 3.74 Å. The angle between thiophenes C and G is higher (18.36°) but the two S-atoms are involved in a weak non-covalent interaction with a distance of 3.81 Å between them. The strong π–π stacking interaction and the presence of multiple S–S non-covalent interactions in the H-shaped TTF-oligothiophene system **54** (*n* = 2) made this compound a promising p-type organic semiconductor material. The time of flight mobility for this compound was found to increase from 1.4 × 10^−6^ to 1.1 × 10^−5^ cm^2^ V^−1^ s^−1^, as the electric field increases from 1 × 10^5^ to 4 × 10^5^ V cm^−1^ [92].

**Figure 6:**
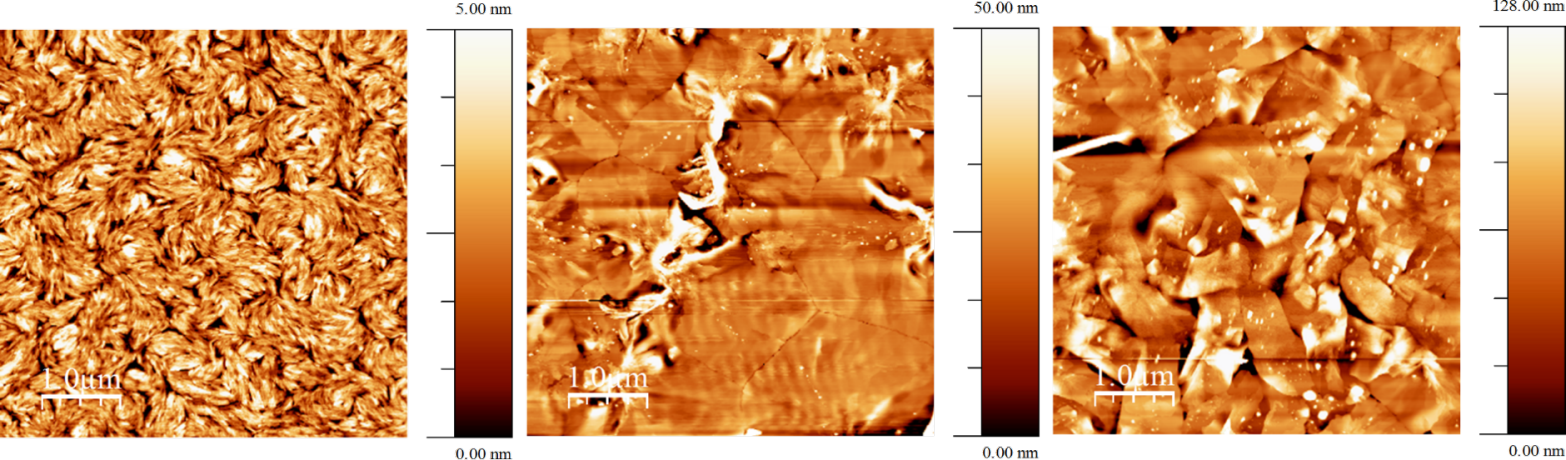
AFM tapping mode images of the compound **54** (*n* = 1) film cast on an untreated SiO_2_ substrate surface from solutions in chlorobenzene (left), CHCl_3_ (centre) and on an ODTS treated SiO_2_ substrate from CHCl_3_ (right). Reproduced with permission from [93]. Copyright 2014 The Royal Society of Chemistry.

Compound **54** (*n* = 1) was tested as a solution processable p-type semiconductor in OFETs using two solvents for spin-coating – chloroform and chlorobenzene [93]. A bottom contact, bottom gate device configuration was used with an n-doped silicon gate and a SiO_2_ dielectric layer. After annealing at 120 °C, AFM imaging indicated a closely packed grain-like surface morphology of the film cast from chlorobenzene as a result of the strong propensity of H-shaped TTF-quinquithiophene **54** (*n* = 1) to aggregate in this solvent (Figure 6, left). Upon spin-coating and further annealing, the rate of nucleation exceeded the rate of grain growth, leading to the small size of the crystalline domain. An OFET mobility of 1.41 × 10^−4^ cm^2^ V^−1^ s^−1^ calculated in the saturation region was observed. An increase in the field effect mobility (to µ = 1.17 × 10^−3^ cm^2^ V^−1^ s^−1^) by almost an order of magnitude was observed in devices cast from chloroform. As with polymer **48**, tapping mode AFM of the organic semiconductor film spin-coated from this solvent revealed that, after annealing, the surface morphology consisted of large crystalline domains with a smooth grain boundary (Figure 6, centre). Such a striking difference in morphology of the films cast from chlorobenzene and CHCl_3_ is explained by the high energy of solvation of **54** (*n* = 1) in chloroform, which leads to a higher crystallisation rate compared to the rate of nucleation during spin-coating and subsequent annealing. When substrates with a pre-treated *n*-octadecyltrichlorosilane (ODTS) SiO_2_ surface were used for spin-coating from a CHCl_3_ solution, the surface morphology remained essentially the same (Figure 6, right), with a further increase in mobility (µ = 8.61 × 10^−3^ cm^2^ V^−1^ s^−1^) observed due to the beneficial effects of large crystalline domains on the field effect mobility.

Compound **54** (*n* = 1) was tested as a donor material in BHJSCs. The results are presented in Table 3. Similar to polymer **48**, the device prepared by spin-coating the blend of **54** (*n* = 1) and PC_71_BM from solution in *o*-DCB showed a higher performance than that fabricated with CHCl_3_, with a short circuit current density (increased up to 9.81 mA cm^−2^) being more affected by the solvent than the open circuit voltage (0.78 V). AFM revealed a smoother surface morphology of the donor–acceptor blend film cast from *o*-DCB than that when chloroform was used as solvent.

## Conclusion

The series of poly- and oligothiophene based compounds bearing TTF units reported so far in the literature have been discussed. The most interesting properties were exhibited by polymers where TTF units were incorporated alongside the conjugated backbone, allowing for the different charge transport mechanisms on the basis of TTF mixed valence states and polarons to be observed. Upon positioning the TTF unit in the vicinity of the polymer backbone, a variation of electrochemical behaviour is observed, including complete dominance by the TTF units and, at times, independent activity of both electroactive entities.

The initial idea of creating materials with hybrid charge transport on the basis of the polaron mechanism and the mixed valence state of doped TTF units has developed now into efforts to use the TTF unit as a handle for controlling the morphology of organic semiconductors in the solid state. The great challenge in this field is to design hybrid materials where the position of the TTFs relative to the polymer backbone and the choice of optimised processing conditions allow tuning of the energy levels and the intrinsic charge carrier mobility in order to achieve maximum device performance.
